# Thermal Diffusivity Mapping of Graphene Based Polymer Nanocomposites

**DOI:** 10.1038/s41598-017-05866-0

**Published:** 2017-07-17

**Authors:** Matthieu Gresil, Zixin Wang, Quentin-Arthur Poutrel, Constantinos Soutis

**Affiliations:** 1 i-Composites lab, School of Materials, University of Manchester, Manchester, 79 Sackville street, M1 3NJ UK; 2 Aerospace Research Institute, University of Manchester, Manchester, 79 Sackville street, M1 3NJ UK

## Abstract

Nanoparticle dispersion is widely recognised as a challenge in polymer nanocomposites fabrication. The dispersion quality can affect the physical and thermomechanical properties of the material system. Qualitative transmission electronic microscopy, often cumbersome, remains as the ‘gold standard’ for dispersion characterisation. However, quantifying dispersion at macroscopic level remains a difficult task. This paper presents a quantitative dispersion characterisation method using non-contact infrared thermography mapping that measures the thermal diffusivity (α) of the graphene nanocomposite and relates α to a dispersion index. The main advantage of the proposed method is its ability to evaluate dispersion over a large area at reduced effort and cost, in addition to measuring the thermal properties of the system. The actual resolution of this thermal mapping reaches 200 µm per pixel giving an accurate picture of graphene nanoplatelets (GNP) dispersion. The post-dispersion treatment shows an improvement in directional thermal conductivity of the composite of up to 400% increase at 5 wt% of GNP. The Maxwell-Garnet effective medium approximation is proposed to estimate thermal conductivity that compare favourably to measured data. The development of a broadly applicable dispersion quantification method will provide a better understanding of reinforcement mechanisms and effect on performance of large scale composite structures.

## Introduction

Graphene consists of a single layer of graphite resulting in high stiffness, superior electrical properties and an exceptionally large thermal conductivity of 3000 to 5000 W/mK in plane at room temperature^1^. Dispersion is the process of de-agglomeration and distribution of nanofillers within matrices or solvents. Since the electro-thermo-mechanical properties of polymer nanocomposites depend on the quality of the dispersion, agglomeration of nanofillers has been reported to lead to property variation over the composite structure^2–6^. The degree of variability is determined directly by the degree of agglomeration. The difficulty of dispersion is due to the small size of graphene nanoplatelets (GNPs), their large surface area and the inherent Van der Walls force between the nanofillers.

Developing a quantitative measurement of dispersion is a challenge that involves systematically studying loading, particle size, agglomerates and interfacial interactions, where transmission electron microscopy (TEM) remains as a gold standard^7^ but restricted to relatively small size samples. Moreover, the majority of these characterisation techniques have been used to assess the nanofillers distribution on a qualitative basis only, providing no means to quantify the extent of distribution. Khare *et al*.^7^ employed TEM with a free-space length method to analyse dispersion, density and length-scale. TEM provides composition and structural information, revealing surface features, shape and size without any physical properties. A lower resolution alternative is the scanning electron microscopy (SEM). Fu *et al*.^8^ introduced a method to quantify the homogeneity of carbon nanotubes (CNTs) in epoxy, where dispersion was evaluated by processing SEM images and the distribution index was calculated by dividing the images into grids. Pfeifer and Bandaru^9^ employed optical microscopy, which correlates randomness with dispersion. However, TEM and SEM are expensive techniques and can only scan across a reduced field of the sample, losing macroscopic-scale information. A myriad of nano-scale and micro-scale quantitative methods have been proposed^4, 6, 7, 9–17^, but none of these are suitable for large scale analysis and representative dispersion characterisation in composite structures. Moreover, these systems are used for low weight percentage of nanoparticles, where in order to be effective which is also not convenient to use for thermal management, where in order to be effective a high weight percentage (5–10%wt) of graphene might be required.

The thermal conductivity of nanocomposites is determined by lattice atomic vibrations (via a pseudo-particle called phonon), which are influenced by the loading weight, the aspect ratio of nanoparticles, the dispersion quality and the interfacial interactions between nanoparticles and matrix^18^. With large weight fractions of graphene and high surface areas it is easy to generate agglomerates due to large Van der Waals force among nanoparticles. This causes phonon mismatching between two phases, increasing interfacial thermal resistance, increasing phonons scattering and so decreasing thermal conductivity^19^. GNP can achieve a uniform dispersion and good thermal conductivity by phonon diffusion through their large platelet morphology^20^. Thermal conductivity does not present a percolation threshold, and it normally increases several times compared to neat epoxy with increasing loading weight^21^. Chandrasekaran *et al*.^22^ found that an increasing GNP content (2%wt) results in a higher thermal conductivity (0.22 W/mK), and observed no percolation behaviour.

However, a large loading increases viscosity of the epoxy which is detrimental to the composite fabrication quality. In this case, nano-fillers with high aspect ratios are necessary to simplify the fabrication process. To achieve large thermal conductivities with a low filler loading, some researchers employed functionalised nanofillers to reduce phonon mismatching between the matrix and the nanoparticles. Kim *et al*.^23^ found that surface-treated GNP reduce interfacial resistance between nanoparticles and the polymer matrix, improving thermal conductivity. Another option is to align nanofillers by an electric field. Martin *et al*.^24^ noticed that inside an epoxy system, the surface of CNT is negatively charged, due to the basic character of the epoxy system. This charge induces an electrophoretic displacement under a DC electric field and hence controls orientation. It was found that the alignment was better under an AC field via the dielectrophoresis induced by the non-constant electric field and the Coulomb interaction between the tips of the CNT. This interaction is due to the higher polarisation of the CNT in the axis direction than the radial direction. Wu *et al*.^25^ have studied the alignment of GNP under an electric field and shows that the same behaviour is observed as that for CNT. Under an AC field the anisotropic shape of GNP induced their rotation along the electric field direction, then the dipole interaction created by the non-constant field leads to an “end-to-end chain” formation. With a controlled network they improved the resin’s thermal conductivity by nearly 60%^26^.

Thermal diffusivity represents the rate of heat conduction through a material. In 1961, Parker *et al*.^27^ developed the flash method where a lamp or laser is used as energy source; it remains the most frequently used transient photo-thermal technique for measuring the thermal diffusivity of solid materials. The laser flash method is described by the American Society for Testing and Materials E-1461 standard^28^. Parker *et al*.^27^ proposed a semi-empirical equation for thermal diffusivity expressed as

1 $${\alpha }_{0.5}=\frac{0.1388{h}^{2}}{{t}_{0.5}}$$

with *h* the thickness of the sample and *t*_05_ is the halftime of the specimen required to reach the maximum temperature. It is assumed that the sample material is homogeneous and isotropic, the heat flow is one dimensional, and there are no heat losses^27^. It also states that the energy pulse is instantaneous and uniformly illuminates the front face of the specimen. In order to avoid these assumptions, Clark and Taylor^29^ examined the thermogram at different points before the maximum temperature was reached and developed a correction factor, (*C*_*R*_):

2 $${C}_{R}=-0.3461467+0.361578(\frac{{t}_{0.75}}{{t}_{0.25}})-0.06520543{(\frac{{t}_{0.75}}{{t}_{0.25}})}^{2}$$

The corrected value for thermal diffusivity becomes

3 $${\alpha }_{corrected}=\frac{{\alpha }_{0.5}{C}_{R}}{0.13885}$$

This method has been used to measure the diffusivity for non-destructive evaluation (NDE) inspecting porosity^30, 31^, bonding defects^30^ and impact damage^32^ in composite laminates. The mapping of the thermal diffusivity requires its calculation for each pixel. In the present study, the mapping can illustrate and quantify the distribution of diffusivity that relates to homogeneity (i.e. the quality) of the GNP dispersion. The resolution of the technique is not able to characterise accurately the dispersion at nanoscale, but more suitable at the micro or macro-scale for a relatively large scale sample. Manufacturing a part of an aircraft with nanofillers can be very useful for several applications, such as lightning strike protection using the superior electro-thermal conductivity of graphene. However, for such structures the quality of the GNP dispersion is a key parameter and will require further work.

The thermal conductivity depends on the acoustic transport of phonon that relates to elastic vibrations of lattice and the heat flux between nanoparticles and the matrix. It is limited by phonon scattering caused by imperfections in the lattice. In nanocomposites, thermal transport depends on the loading weight, the dispersion quality, the structure of the nanoparticle and the thermal contact resistance between the particle and the matrix. The thermal conductivity, *K*, is calculated by

4 $$K=\rho \alpha {C}_{p}$$

where *ρ* denotes the density of the sample, *α* is the thermal diffusivity and *Cp* is the heat capacity at constant pressure. Here the density is measured by a Mettler Toledo machine were samples are dried at 80 °C for at least 24 hours. The heat capacity, *C*_*p*_, is measured by the differential scanning calorimeter (DSC) at constant pressure and is given by

5 $${C}_{P}=\frac{Total\,heat\,flow}{Average\,heating\,rate}\times c$$

where *c* is a calibration constant.

In this paper, an effective method of mapping thermal diffusivity is developed and a dispersion index is established to quantify this over a large area. A post-dispersion treatment, based on alignment of GNP using electrical field, is developed to enhance the thermal conductivity, *k*, of the composite. The Maxwell-Garnet effective medium approximation is proposed to estimate *k* and this is validated with thermal experimental data.

## Results

### Thermal conductivity and diffusivity of the non-align GNP nanocomposites

**Figure 1:**
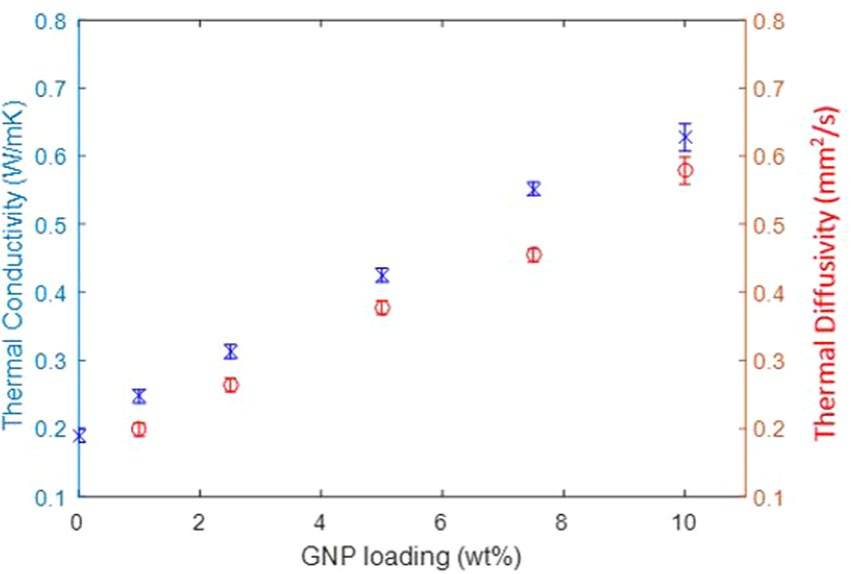
Thermal conductivity and diffusivity of the nanocomposite vs GNP loading fraction.

The average values of density and heat capacity are shown in Table 1; four samples for each GNP loading were tested in this study. The data show that density and heat capacity are almost independent of GNP loading, especially for more than 2.5 wt% GNP. The thermal conductivity and diffusivity are plotted versus the GNP loading in Fig. 1, and a linear relationship is observed:

6 $$K=\rho {C}_{p}\alpha \approx 1.21\pm 0.01\times \alpha $$

**Table 1:** Average measured values of density and heat capacity.

Samples (wt%)	Density (g/cm^3^)	Heat capacity (J/gK)
1	$1.13\pm 0.02$	$1.078\pm 0.01$
2.5	$1.12\pm 0.02$	$1.082\pm 0.01$
5	$1.14\pm 0.02$	$1.086\pm 0.01$
7.5	$1.13\pm 0.02$	$1.086\pm 0.01$
10	$1.13\pm 0.02$	$1.086\pm 0.01$

Thermal diffusivity indicates the speed of heat transfer in the sample, and it correlates well with the thermal conductivity so the terms can be considered interchangeable. The thermal diffusivity was obtained from the infrared thermography measurement in conjunction with equation 3, while equation 5 was used to calculate the thermal conductivity. At 10 wt% GNP, the thermal conductivity is 0.63 ± 0.01 W/mK which represents an improvement of 331% when compared to pure epoxy (0.19 ± 0.06 W/mK).

It should be noted that the value of *K* depends very much on the density and heat capacity measurements that employ small size (less than 50 mg) samples and for statistically valid data (affected by dispersion) would require an extensive number of specimens. A small area of the sample cannot represent the heat capacity of the whole nanocomposite plate especially if there is agglomeration and other fabrication induced imperfection which cause heterogeneity.

**Figure 2:**
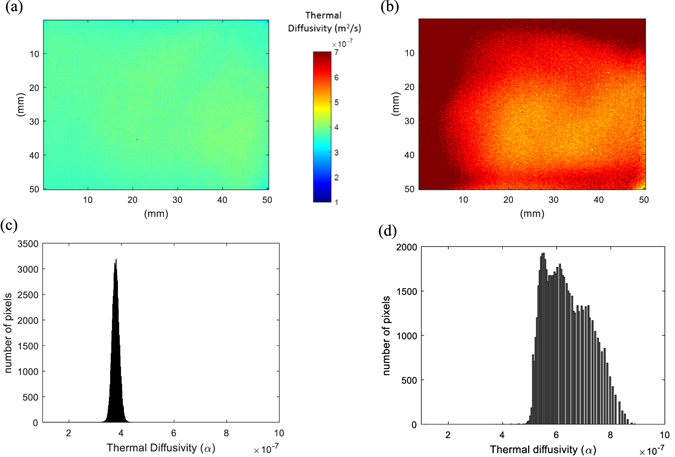
Thermal diffusivity mapping of (**a**) 5.0 wt%, and (**b**) 10.0 wt% GNP based epoxy nanocomposites. Number of pixel vs thermal diffusivity which represents the degree of dispersion (standard deviation); (**c**) 5 wt%; (**d**) 10 wt% of GNP.

It is interesting to note in Fig. 1 that there is no indication of thermal percolation threshold for the samples examined, but this may change for higher amounts of nanoplatelets. However, this would increase the viscosity of the mixture and contribute to the aggregation of GNP that damages dispersion, even at increased shear mixing temperature. Agglomeration in the material influences phonon transportation that affects homogeneity of thermal conductivity (Fig. 2). In the following section, the thermal diffusivity measured by IR thermography is related quantitatively to a dispersion index.

### Thermal diffusivity mapping and dispersion index

Figure 2a and Fig. 2b illustrate the thermal diffusivity of 5.0 and 10.0 wt% GNP based epoxy nanocomposites. Through this simple, non-contact and cost-effective technique, the thermal diffusivity is mapped. The scale bar ranges from a value of 1 · 10^−7^m^2^s^−1^ to 7 · 10^−7^m^2^s^−1^ where darker (red) the picture, higher the thermal diffusivity is; spatial resolution for one pixel is 200 μm. This can be improved by (i) varying the position of the infrared camera close to the sample; (ii) using a magnifying lens; or (iii) employing a high definition camera. Colour differences in the sample reflect the quality of dispersion of nanoparticles. Strong colour variations indicate a larger thermal heterogeneity. In Fig. 2a (5.0 wt% GNP) the measured thermal diffusivity is more uniform compared to that observed in Fig. 2b for 10 wt% GNP composite where nanoparticles agglomeration may be present.

To quantify and characterise the dispersion quality through IR thermography, the standard deviation of the thermal diffusivity of pixels in the image is investigated. Figure 2c,d indicate the distribution of thermal diffusivity data of each pixel. The x-axis shows the thermal diffusivity value from 3 · 10^−7^m^2^/s to 9 · 10^−7^m^2^/s, while y-axis is the number of pixels. When the distribution is narrow as that shown for 5.0 wt% GNP in Fig. 2c, it corresponds to a homogeneous dispersion, unlike the 10 wt% GNP, Fig. 2d, where the distribution curve is wide and varied (i.e. non uniform thermal diffusivity).

A dispersion index (DI) is then calculated to quantify the quality of the dispersion/homogenisation of the GNP in a large nanocomposite plate. This is defined as:

7 $$DI=\frac{{\alpha }_{\max }(100pixel)-{\alpha }_{\min }(100pixel)}{{\alpha }_{peak}}$$

where ${\alpha }_{\max }(100pixel)$ and ${\alpha }_{\min }(100pixel)$ represent the maximum and the minimum of the thermal diffusivity at 100 pixels, respectively, while ${\alpha }_{peak}$ is its peak value. The value of 100 pixels is taken arbitrarily, as a reference. DI varies between 0 and 1, and for a narrow band DI approaches 0 suggesting a uniform GNP dispersion.

**Figure 3:**
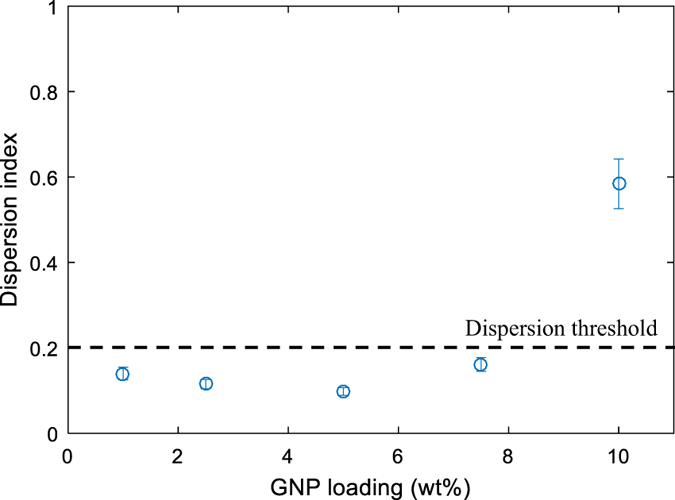
Dispersion index (DI) vs GNP loading based on equation (7).

Figure 3 presents the dispersion index versus the GNP loading in wt%. The higher value of DI is approximately 0.6 and corresponds to 10 wt% GNP where the reinforcing platelets appeared not to be well dispersed as illustrated in Fig. 2b. For all the other GNP loadings, the DI is almost constant with a value of less than 0.2 which can be considered as a threshold value. Above this threshold, dispersion is not optimal although the local thermal conductivity may be high, but not uniform across the whole specimen. It is suggested that this threshold value could be used to obtain a GNP nanocomposite with enhanced and homogeneous thermal properties.

### Thermal conductivity the aligned nanocomposite

**Figure 4:**
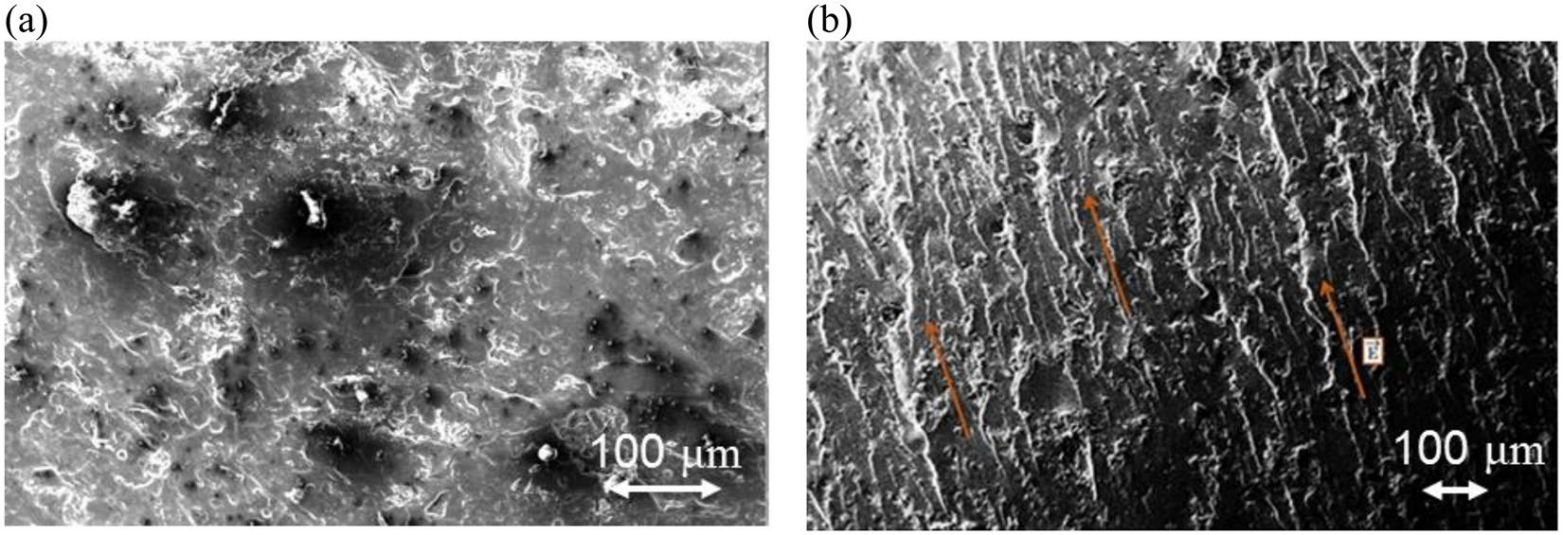
SEM images of fracture surface: (**a**) of non-aligned GNP based epoxy composite (1 wt%): GNP show independent direction and no specific connection between each other; (**b**) of aligned GNP based epoxy composite (1 wt%): chain of GNP through the composite along the applied electric field.

**Figure 5:**
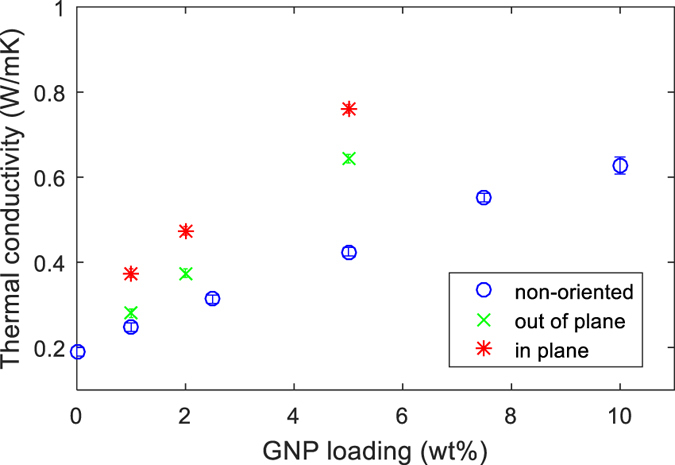
Thermal conductivity vs GNP loading for non-oriented, oriented parallel and perpendicular to the applied electric field direction.

**Figure 6:**
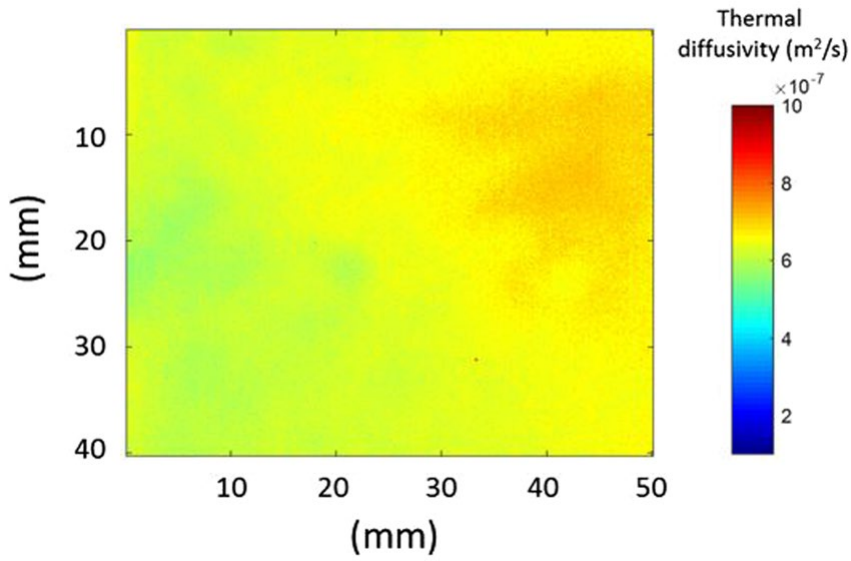
Thermal diffusivity mapping for the aligned 5 wt% GNP panel.

In order to improve the nanocomposites properties after the dispersion and before the curing, the nanoparticles are aligned using an electrical field as described in section 3. The SEM image of the fracture surface of the 1 wt% GNP nanocomposite without the application of the electric field is shown in Fig. 4a. As expected, the GNPs are randomly oriented and distributed, of different size and shape. Figure 4b shows the microstructure of the same system after the electric field applied. Most of the platelets are well aligned and parallel to the AC electric field direction. The dipole interaction (dielectrophoresis) created by the non-constant field leads to an “end-to-end chain” formation of GNP networks allowing a better heat flow through the nanocomposite. Voids are also observed in both cases which are developed during the mixing and curing process which can decrease the phonon transportation. Figure 5 shows the evolution of thermal conductivity with the GNP loading for non-oriented, oriented parallel and perpendicular to the applied electric field direction. The thermal conductivity of 5 wt% GNP loading along the direction of the applied field is $K=0.76W/\mathrm{mK}$, while for the randomly oriented is $K=0.42W/\mathrm{mK}$. This 180% improvement confirms the alignment of the particles. This alignment provides an “easy” path for the phonons to travel (vibrate) resulting in better thermal conductivity. Figure 6 illustrates the thermal diffusivity of the aligned sample (5 wt% GNP) with DI = 0.16 which is below the defined dispersion threshold of 0.2 confirming a homogeneous distribution of GNP.

This alignment experiment demonstrates that the post-dispersion step during the manufacturing process has a large influence on the thermal properties of the composite. It should be noted that several other parameters such as type of particles (size and shape), temperature, viscosity, and GNP loading can have an impact on the thermal response and need to be carefully considered in the design of any composite system.

### Theoretical value of thermal conductivity

In this section, the Maxwell-Garnett effective medium approximation (MG-EMA) is employed to better understand the experimental data discussed earlier. The effective thermal conductivity of the composite with GNP particles (valid for less than 40% vol) is given by refs 33 and 34.

8 $${K}_{11}^{\ast }={K}_{22}^{\ast }={K}_{m}\frac{2+\varphi [{\beta }_{11}(1-{L}_{11})(1+\langle {\cos }^{2}\theta \rangle )+{\beta }_{33}(1-{L}_{33})(1-\langle {\cos }^{2}\theta \rangle )]}{2-\varphi [{\beta }_{11}{L}_{11}(1+\langle {\cos }^{2}\theta \rangle )+{\beta }_{33}{L}_{33}(1-\langle {\cos }^{2}\theta \rangle )]}$$

9 $${K}_{33}^{\ast }={K}_{m}\frac{1+\varphi [{\beta }_{11}(1-{L}_{11})(1-\langle {\cos }^{2}\theta \rangle )+{\beta }_{33}(1-{L}_{33})\langle {\cos }^{2}\theta \rangle ]}{1-\varphi [{\beta }_{11}{L}_{11}(1-\langle {\cos }^{2}\theta \rangle )+{\beta }_{33}{L}_{33}\langle {\cos }^{2}\theta \rangle ]}$$

With

10 $${\beta }_{ii}=\frac{{K}_{ii}^{c}-{K}_{m}}{{K}_{m}+{L}_{ii}({K}_{ii}^{c}-{K}_{m})}$$

11 $$\langle {\cos }^{2}\theta \rangle =\frac{\int \rho (\theta ){\cos }^{2}\theta \,\sin \,\theta \,d\theta }{\int \rho (\theta )\sin \,\theta \,d\theta }$$

where *θ* is the angle between the material axis *X*_3_ and the local particle symmetric axis ${X^{\prime} }_{3}$, *ρ*(*θ*) is a distribution function describing ellipsoidal particle orientation, *ϕ* is the volume fraction of particles, ${K}_{ii}^{c}(i=1,2,3)$ are the equivalent thermal conductivities along symmetric axis of the composite unit cell, *K*_*m*_ is the thermal conductivity of the matrix phase and *L*_*ii*_ are geometrical factors dependent on the particle shape given by ref. 26

12 $$\begin{array}{c}{L}_{11}={L}_{22}=\frac{{r}^{2}}{2({r}^{2}-1)}-\frac{r}{2{(1-{r}^{2})}^{3/2}}{\cos }^{-1}r,{\rm{for}}\,r < 1\\ {L}_{33}=1-2{L}_{11}\end{array}$$

where *r* = *a*_3/_ *a*_1_ is the aspect ratio of the ellipsoid, and r < 1 is for an oblate ellipsoidal inclusion. When the interfacial thermal resistance is thought of as the limiting case of heat transport across bulk phase separated by a thin, poorly conducting interphase region, the equivalent thermal conductivities ${K}_{ii}^{c}(i=1,2,3)$ is ref. 34

13 $${K}_{ii}^{c}={K}_{p}/(1+\gamma {L}_{ii}{K}_{p}/{K}_{m})$$

With

14 $$\gamma =(1+2r)\delta ,{\rm{for}}\,r\le 1$$

where *K*_*p*_ is the thermal conductivity of the particle, and *δ*, a dimensional parameter, is introduced and defined by:

15 $$\delta ={a}_{k}/{a}_{3},{\rm{for}}\,r\le 1$$

In which the interfacial thermal property is concentrated on a surface of zero thickness and characterised by the Kapitza radius, *α*_*k*_, defined as

16 $${a}_{k}={R}_{Bd}{K}_{m},with\,{R}_{Bd}=\,\mathrm{lim}\,\mathop{\mathrm{lim}}\limits_{\begin{array}{c}\delta \to 0\\ {K}_{s}\to 0\end{array}}(\delta /{K}_{s})$$

with *δ* is the thickness of the surrounding interface layer and *K*_s_ its conductivity.

**Figure 7:**
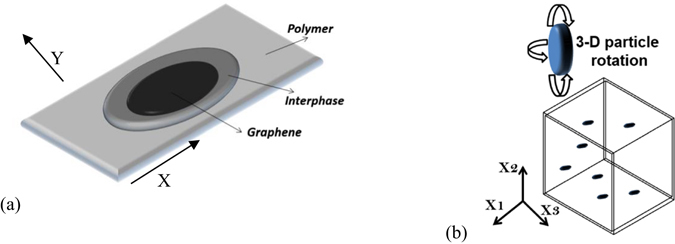
(**a**) Schematic of the unit cell of the graphene surrounded by polymer with the interphase (interfacial thermal resistance); (**b**) Representative volume element with the random distribution of the graphene into the polymer.

The interfacial thermal resistance is known as the Kapitza resistance, *R*_*Bd*_, after Kapitza’s discovery of temperature discontinuity at the metal-liquid interface^35–37^ (Fig. 7). It is due to the scattering of energy carriers (i.e. phonons) at the interface and the mismatch in vibrational spectra of different materials. In graphene, heat transfer is mainly conducted by the acoustic phonons while the contribution of electrons to thermal conductivity is negligible^38^. When a phonon attempts to cross the graphene-polymer interface, it will scatter at the interface, due to the mismatch in their vibrational spectra.

Considering randomly oriented GNP inclusions into the matrix, $\langle {\cos }^{2}\theta \rangle =1/3$ then equations (8) and (9) can be reduced to ref. 34:

17 $${K}^{\ast }={K}_{m}\frac{3+\varphi [2{\beta }_{11}(1-{L}_{11})+{\beta }_{33}(1-{L}_{33})]}{1-\varphi [{\beta }_{11}{L}_{11}+{\beta }_{33}{L}_{33}]}$$

For the GNP nanocomposite (assuming ideal case) *r* → 0, *L*_11_ → 0, *L*_33_ → 1

13 $${K}_{ii}^{c}={K}_{p}/(1+\gamma {L}_{ii}{K}_{p}/{K}_{m})$$

19 $${\beta }_{11}=({K}_{p}-{K}_{m})/{K}_{m},\,\,\,\,\,\,\,\,\,{\beta }_{33}=(1-{a}_{k}/{a}_{3})-{K}_{m}/{K}_{p}$$

Substituting equations (18) and (19) in equation (17), we obtain

20 $${K}^{\ast }={K}_{m}\frac{3+2\varphi [({K}_{p}-{K}_{m})/{K}_{m}]}{3-\varphi [(1-\delta )-{K}_{m}/{K}_{p}]}$$

**Figure 8:**
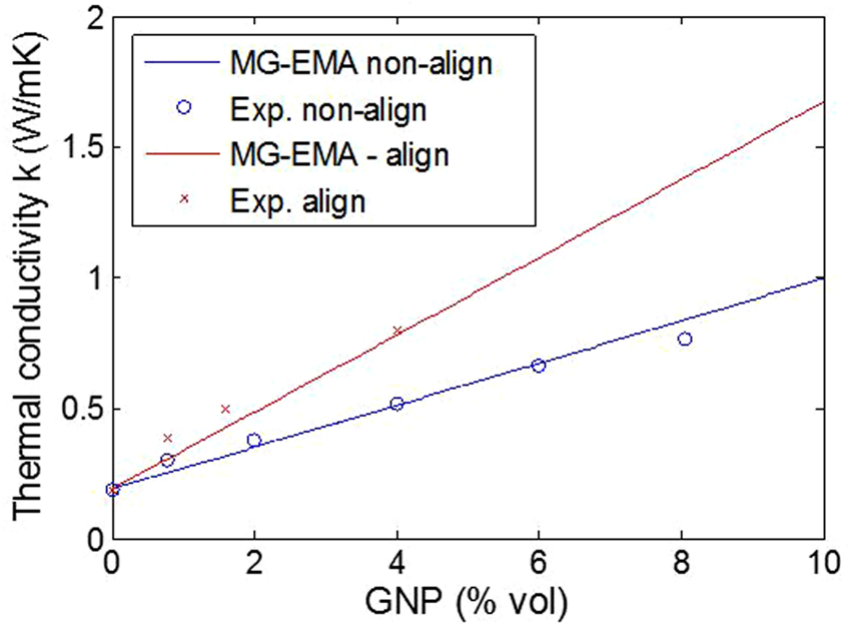
Experimental thermal conductivity fitted with the Maxwell-Garnett model for randomly oriented GNP with Kp = 12 W/mK and align GNP with Kp = 22 W/mK with an interfacial thermal resistance of R_BD_ = 2.10^−8^ m^2^ K/W.

The thermal diffusivity obtained by equation (19) is plotted in Fig. 8 as a function of GNP loading. An average value is assumed for the Kapitza resistance of ${R}_{Bd}=2\times {10}^{-8}{{\rm{m}}}^{{\rm{2}}}K/W$ ^39^, the GNP thermal conductivity is ${K}_{p}=12W/\mathrm{mK}$ for the randomly oriented, and ${K}_{p}=22W/\mathrm{mK}$ for the sample with aligned particles. These values are very small when compared to the thermal conductivity of the pure single layer graphene (~3000 W/mK). This difference may come from the fact that the phonon scattering in the nanocomposite occurs in the out-of-plane direction (through the thickness) of the graphene platelet (see Fig. 7a – Y direction) and also the particles used here consist of 18 layers or more that can be considered as amorphous carbon (with *K*~6 W/mK). This model assist us to identify the actual value of *K*_*p*_ for graphene when is mixed to the resin. Figure 8 shows a good agreement between the model and experiment but there are several simplifications and uncertainties in the analysis and the most important is the assumed value of the particle thermal conductivity. Others assumptions such as monodisperse elliptical particles, perfectly isotropic and uniformly dispersed with no fabrication induced defects can have an impact on predicted results.

## Conclusion

The infrared thermography diffusivity measurement and mapping method presented in this paper has been validated to be a reliable, easy and low cost technique to characterise the quality of graphene nanoplatelets dispersion at macroscale for relatively large samples. The actual resolution of this technique reaches 200 µm and can be easily improved by using high magnification lens, opening a way to characterise nanocomposite at the microscale. Moreover, a dispersion index was identified to quantify the graphene dispersion in the polymer matrix. A linearly increasing trend in the thermal conductivity has been confirmed for up to 10 wt% GNP loading weight. The post-dispersion treatment (i.e. alignment of GNP using electrical field) shows an improvement in directional thermal conductivity of the 5 wt% GNP composite of up to 400% increase. This anisotropic behaviour could be suitable for specific application such as de-icing or lightning strike protection in aircraft industry.

Unlike other dispersion quality quantification and thermal diffusivity mapping techniques, IR thermography is unique due to its ability of mapping large scale samples in an efficient and less cumbersome way.

Currently, interfacial thermal resistance at the graphene/polymer interface is one of the key barrier in further improving the thermal conductivity of graphene based polymer nanocomposites as described in the modelling section. Recent research studies have shown that covalent and non-covalent functionalisation techniques are promising in reducing the interfacial thermal resistance to achieve superior thermal conductivity. Further in-depth research studies are needed to explore the mechanisms of thermal transport across the graphene/polymer interface.

## Methods

### Materials

**Figure 9:**
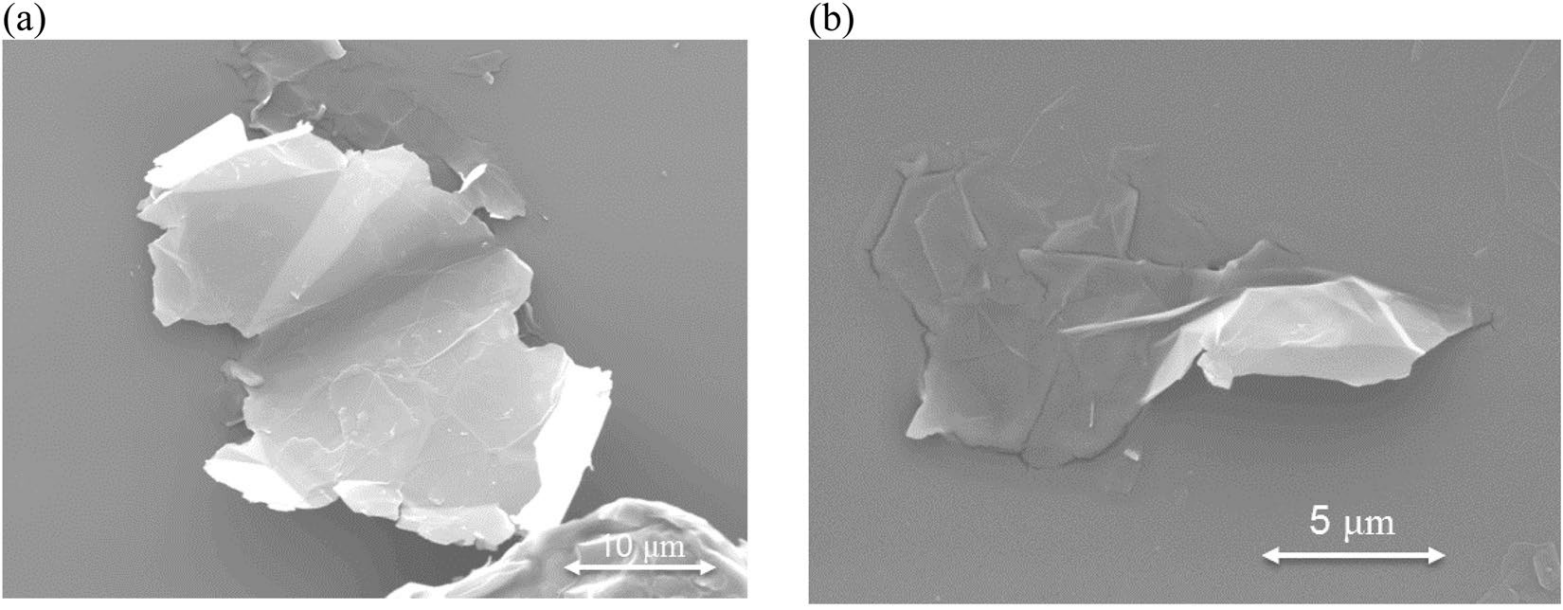
(**a**) SEM image of GNP revealing overlapping regions and wrinkles, which also can improve thermal coupling. (**b**) SEM image of a large flake extracted from the polymer solution after dispersion.

Grade M25 × GnP® graphene nano-platelets were acquired from XG Sciences, consisting of short stacks of graphene sheets on a powder form. The average thickness of 18 layers of graphene is 6–8 nm, and the typical surface area is 120 to 150 m^2^/g with an average length of 25 μm as shown in the SEM image, Fig. 9a. The polymer matrix contains a low-viscosity bisphenol-A epoxy resin (Araldite®LY564) and a cycloaliphatic polyamine curing agent (Aradur®2954) with a mixing ratio of 100:35 (epoxy: hardener) supplied by Huntsman (Switzerland).

### Sample preparation

Graphene nanoplatelets were added to the preheated polymer matrix at 120 °C and shear mixed (SilverSon L5MPA) at 2000 rpm for 2 hours at 80 °C with loading weights ranging from 0.5 to 10 wt%^40^. Then the mixture was cooled down to room temperature over 30 minutes. Figure 9b shows a SEM image of a large flake extracted from the resin solution where its length due to mixing has been reduced down to approximately 12 μm.

**Figure 10:**
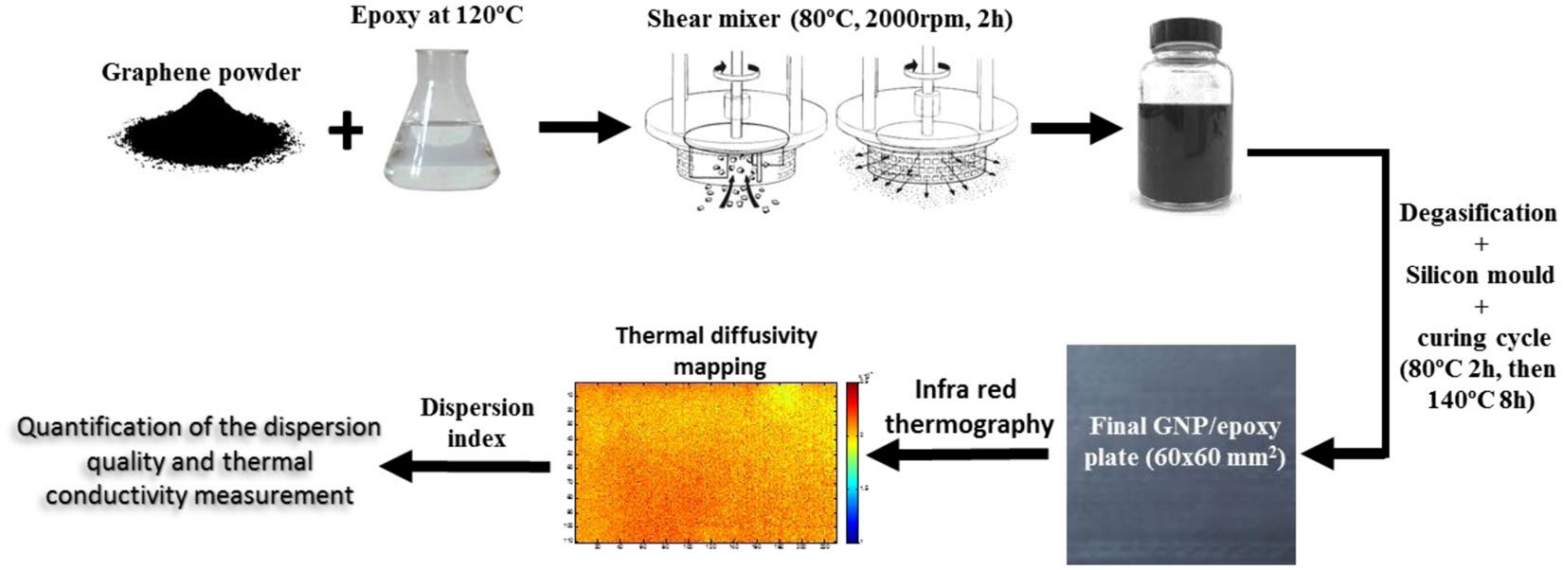
Schematic of the experimental dispersion and characterisation using the IR thermography technique.

After the addition of hardener, the mixture was mechanically stirred for 3 minutes at 1000 rpm. Degasification followed and the mixture was poured into a silicon mould. The nanoparticles were aligned through the plate thickness using an electrical field of 11 kV/m^41^. This is achieved with a square signal at 100 kHz with an amplitude of 1000 Vpp. The samples are cured at 80 °C for 2 hours and post-cured at 140 °C for 8 hours. Figure 10 shows a typical 60 × 60 mm^2^ GNP plate; 2 and 5 mm thick plates were made.

### Thermal characterisation

The thermal diffusivity, α, of the nanocomposite samples is measured and mapped using infrared (IR) thermography. An external heat source (two flash lamps, delivering a combined pulse of approximately 6 kJ for a duration of 10.6 ms) heats the front face of the sample while an IR camera (Thermosensorik GmbH, Germany) records the thermal radiation (temperature) of the rear surface of each pixel (transmission mode). Every IR image consists of 400 × 400 pixels, and the thermal diffusivity of each pixel is calculated. The resolution of the pixel is 150 µm.

Heat capacity is obtained using A Q100 differential scanning calorimeter (DSC) in modulated mode (−50 °C to 100 °C to −50 °C). Cured samples of less than 50 mg in weight were processed in the DSC. The calorimeter was calibrated by a sapphire disk with an empty pan as the baseline. Two heating rates were used simultaneously: one is to provide total heat flow rate at 3 °C per min, and the other is used to measure the heat capacity of the sample with heating rate of 1 °C per min (Eq. 4).

Figure 10 summarises the manufacturing process and the thermal diffusivity mapping for the quantification of the dispersion characterisation.
